# Pharmacokinetics, Tissue Distribution and Excretion of Verticinone from *F.* *hupehensis* in Rats

**DOI:** 10.3390/molecules191220613

**Published:** 2014-12-10

**Authors:** Xiao Wu, Jian-Guo Sun, Ying Peng, Yan Liang, Guang-Ji Wang, Hui Chen, Ji-Zhou Wu, Peng Zhang

**Affiliations:** 1 Hubei Key Laboratory of Natural Medicinal Chemistry and Resource Evaluation, School of Pharmacy of Tongji Medical College, Huazhong University of Science and Technology, 13# Hangkong Road, Wuhan 430030, China; E-Mails: wuxiao_1983@163.com (X.W.); chinahuichen@hotmail.com (H.C.); ywjz@mails.tjmu.edu.cn (J.-Z.W.); 2 State Key Laboratory of Natural Medicines, Key Laboratory of Drug Metabolism and Pharmacokinetics, China Pharmaceutical University, 24# Tongjiaxiang, Nanjing 210009, China; E-Mails: jgsun_cpucn@yahoo.com.cn (J.-G.S.); rose.pearl@yahoo.com.cn (Y.P.); YanLiang@163.com (Y.L.); 3 Tianjin Key Laboratory of Quality Control in Chinese Medicine, Tianjin Zhongxin Pharmaceuticals R&D Center, 21# 10th Avenue, Tianjin 300457, China

**Keywords:** verticinone, pharmacokinetics, tissue distribution, excretion, *F. hupehensis*, Liliaceae

## Abstract

Verticinone, the main active component in *F. hupehensis*, exhibits potent antitussive and expectorant effects. Here, a LC-MS method was developed and applied to study the pharmacokinetics, tissue distribution and excretion of verticinone in rats, and its plasma protein binding* in vitro*. A significant gender difference in the pharmacokinetics of verticinone in rats was observed, as its absolute oral bioavailability in male and female rats was 45.8% and 2.74%, respectively. The relative bioavailability of verticinone was significantly lower in female rats as compared to male, following intragastrical (*i.g.*) and intravenous (*i.v.*) administration. After successive *i.g.* administration of verticinone, accumulation was observed in female rats but not in the male ones. The tissue distribution study showed that verticinone had a good tissue penetrability and a high tissue affinity in most studied tissues, except brain. After a 2 mg/kg oral dose, less than 4% of the dose was excreted as unchanged parent compound in male rats, and less than 1% in female rats, which indicated that verticinone was metabolized more extensively in female rats than in male rats.

## 1. Introduction

**Figure 1:**
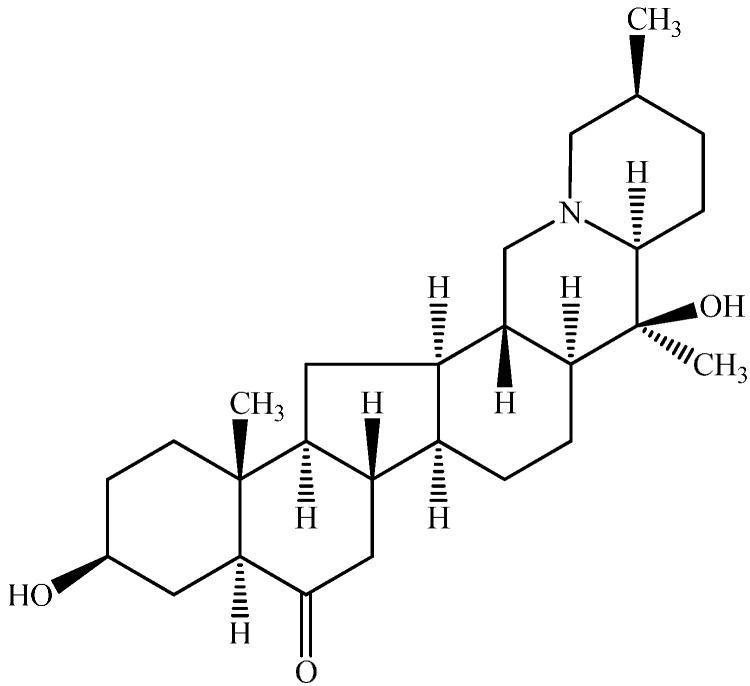
Chemical structure of verticinone.

Beimu, the dried bulbs of various *Fritillaria* species, has been used in Traditional Chinese Medicine as an important antitussive and expectorant herb for more than 2000 years. The pharmacological studies of Beimu extracts have indicated that alkaloids are the main active components [1,2,3,4]. Verticinone (Figure 1) is the main alkaloid in *F. hupehensis* Hsiao *et* KC Hsia (Hubei Beimu), with a content of about 0.3% in the crude herb. It has potent antitussive and expectorant activities [5], and no addictive effect in mice [6,7]. Besides, verticinone also has antitumor [2,8] and analgesic effects [7]. Due to the potent antitussive activity and no addictive effect, verticinone seems to be a promising potential antitussive drug.

During recent years, a lot of work and efforts have been done for the drug development of verticinone in our group. Related reports, including the antitussive mechanism, structural modification and derivation [9,10], quantification analysis in rat plasma [11] have been published. Additionally, guided by the “combination principle” in drug discovery, novel salts and esters of verticinone and bile acids have been synthesized in our lab, which are the major bioactive components in a commercial drug, Shedan-Chuanbei powder [12]. The bioactivity screening studies have shown that both cholic acid-verticinone salt and ester have more potent antitussive activity than verticinone. A preliminary pharmacokinetic study of cholic acid-verticinone ester in rats has been performed in our lab, and no cholic acid-verticinone ester was detected in plasma after *i.g.* administration. Interestingly, when cholic acid was administered in an equivalent dose to cholic acid-verticinone ester to rats, no antitussive effect was observed, which implies that cholic acid-verticinone may act as a verticinone prodrug. To illustrate the more potent activity of cholic acid-verticinone from the perspective of pharmacokinetic behavior, it is imperative to understand the pharmacokinetic behavior of verticinone. As one of the active components in crude extract of *Fritillaria thunbergii*, the pharmacokinetics of verticinone in rats has been studied after the crude extract was administered [13]. Considering the complexity of the crude extract, the potential interactions among different ingredients may influence the pharmacokinetic behavior of verticinone. Therefore, in this report the pharmacokinetics, tissue distribution, excretion and bioavailability following intragastrical administration of verticinone to rats, along with* in vitro* plasma protein binding were investigated.

## 2. Results and Discussion

### 2.1. Pharmacokinetic Study

**Figure 2:**
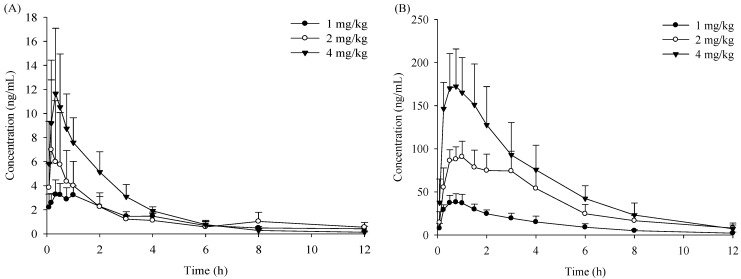
Time-concentration profiles of verticinone after oral dose of 1, 2 and 4 mg/kg in rats. (**A**) female rats; (**B**) male rats.

**Figure 3:**
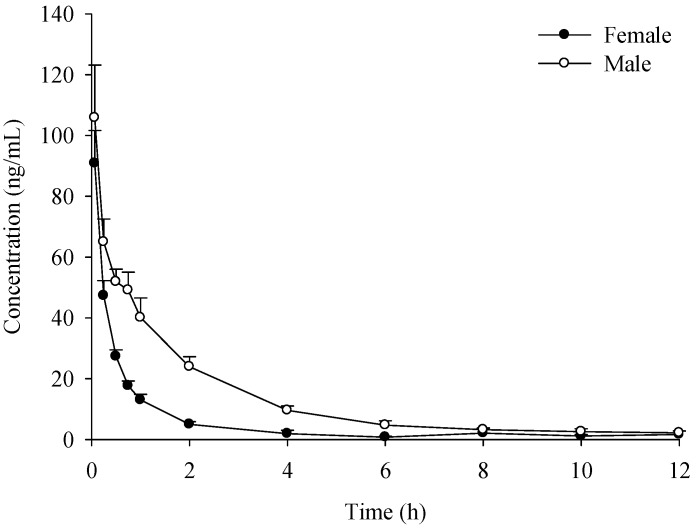
Time-concentration profiles of verticinone after* i.v.* administration of 0.4 mg/kg.

The time-concentration curves of verticinone following *i.g.* (intragastrical) and *i.v.* (intravenous) administration are shown in Figure 2 and Figure 3. The curves fitted well a two-compartmental model after *i.g.* dose and a three-compartmental model after *i.v.* dose based on the Akaike information criterion (AIC). The pharmacokinetic parameters are summarized in Table 1. After the rats were orally administered 1, 2, and 4 mg/kg of verticinone, the relationship between dosages and AUC_0__–t_ of verticinone in plasma showed a linear dependence in male rats, whereas a nonlinear dependence was seen in female rats. The apparent volumes of distribution (Vd) for verticinone were much larger than the total body water in rats [14], indicating that the compound was extensively distributed into extravascular tissues.

**Table 1:** Pharmacokinetic parameters of verticinone after* i.g.* and* i.v.* administration to SD rats (n = 5).

Para-Meters	Unit	*i.g.*						*i.v.*	
		1 mg/kg (Mean ± S.D.)		2 mg/kg (Mean ± S.D.)		4 mg/kg (Mean ± S.D.)		0.4 mg/kg (Mean ± S.D.)	
		♀	♂	♀	♂	♀	♂	♀	♂
C_max_	μg/L	3.67 ± 1.13	38.9 ± 9.34 **	7.45 ± 7.10	94.8 ± 17.1 **	12.6 ± 5.14	176 ± 45.2 **	-	-
T_max_	h	0.67 ± 0.31	0.70 ± 0.11	0.40 ± 0.36	1.45 ± 1.42	0.40 ± 0.35	0.70 ± 0.11	-	-
AUC_0–t_	μg·h/L	14.5 ± 3.27	159 ± 50.1 **	17.3 ± 5.40	446 ± 111 **	26.6 ± 5.84	737 ± 236 **	65.6 ± 7.03	159.5 ± 20.2 **
AUC_0–∞_	μg·h/L	15.3 ± 3.06	167 ± 52.5 **	18.8 ± 5.01	475 ± 119 **	27.1 ± 5.78	769 ± 257 **	98.9 ± 35.8	167.6 ± 23.0 **
MRT_0–t_	h	3.59 ± 0.18	3.21 ± 0.34	3.42 ± 1.22	3.62 ± 0.37	2.04 ± 0.21	3.19 ± 0.48	2.04 ± 0.34	2.37 ± 0.14
t_1/2z_	h	2.86 ± 1.29	2.78 ± 0.85	4.05 ± 2.48	3.05 ± 0.78	1.84 ± 0.58	2.33 ± 0.86	-	-
CLz/F	L·kg/h	67.0 ± 11.6	6.53 ± 2.35 **	113 ± 33.5	4.40 ± 0.98 **	152 ± 28.2	5.59 ± 1.48 **	4.47 ± 1.48	2.43 ± 0.37 *
Vz/F	L/kg	280 ± 160	25.5 ± 9.07 *	649 ± 419	19.4 ± 6.70 **	387 ± 74.2	18.6 ± 9.14 **	82.5 ± 70.3	10.9 ± 1.56

* p < 0.05; ** p < 0.01 (*vs*. female).

Exposure of verticinone was approximately 11~28 fold higher in male rats than in female rats for all three* i.g.* doses and 2.4 fold higher for the* i.v.* dose; the CL and Vd values were much lower in male rats compared with female rats, which indicated a gender difference in the pharmacokinetics of verticinone ocurred in rats. The absolute bioavailability values of verticinone in male and female rats were 45.8% and 2.74%, respectively. The gender difference of pharmacokinetic parameters of verticinone indicated that the compound has different absorption and/or metabolic behaviors in different sex rats.

**Figure 4:**
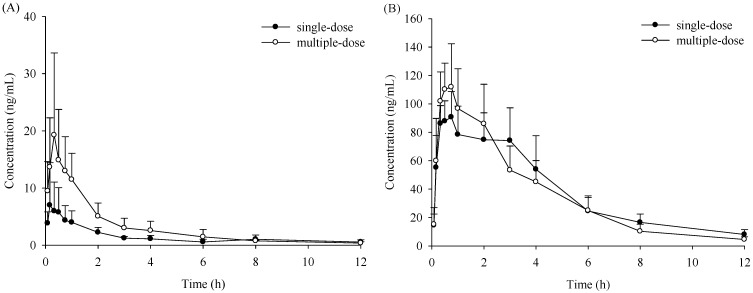
Time-concentration profiles of verticinone after single and multiple-oral dose of 2 mg/kg administration to rats: (**A**) female; (**B**) male.

The time-concentration curves of verticinone after successive *i.g.* administration of 2 mg/kg compared with a single* i.g.* are shown in Figure 4 and the pharmacokinetic parameters are shown in Table 2. After successive *i.g.*, the pharmacokinetic behavior of verticinone also exhibited a significant gender difference. The C_max_ and AUC of the compound were significantly smaller in female rats than that in male rats, which coincides with the finding that the CL was much higher in female rats compared with male rats. For the female rats, C_max_ and AUC were approximately 2-fold higher, and CL was 2-fold lower after successive *i.g.* than single *i.g*. However, no significant difference was found in male rats. Since female rats have lower bioavailability than male rats, the increase of C_max_ and AUC, and the decrease of CL in female rats after successive administration might be caused by a saturated verticinone metabolism, or a metabolism suppression caused by verticinone.

**Table 2:** Pharmacokinetic parameters of verticinone after single and successive *i.g.* administration of 2 mg/kg to SD rats (*n* = 5).

Paramters	Unit	♀ (Mean ± S.D.)		♂ (Mean ± S.D.)	
		Single *i.g.*	Successive *i.g.*	Single *i.g.*	Successive *i.g.*
C_max_	μg/L	7.45 ± 7.10	19.9 ± 14.0	94.8 ± 17.1	119 ± 26.8 ^b^
T_max_	h	0.40 ± 0.36	0.38 ± 0.22	1.45 ± 1.42	0.80 ± 0.21 ^b^
AUC_0–t_	μg·h/L	17.3 ± 5.40	36.1 ± 15.2 ^a^	446 ± 111	439 ± 126 ^b^
AUC_0–∞_	μg·h/L	18.8 ± 5.01	43.4 ± 12.0 ^a^	475 ± 119	455 ± 125 ^b^
MRT_0–t_	h	3.42 ± 1.22	2.24 ± 0.28	3.62 ± 0.37	2.98 ± 0.30 ^b^
t_1/2z_	h	4.05 ± 2.48	5.94 ± 4.84	3.05 ± 0.78	2.08 ± 0.51
CLz/F	L·kg/h	113 ± 33.5	49.0 ± 13.1	4.40 ± 0.98	4.71 ± 1.47 ^b^
Vz/F	L/kg	649 ± 419	485 ± 499	19.4 ± 6.70	13.8 ± 4.07

^a^ Significantly different (*p* < 0.05) from single *i.g.*; ^b^ Significantly different (*p* < 0.01) from female rats.

### 2.2. Tissue Distribution Study

**Figure 5:**
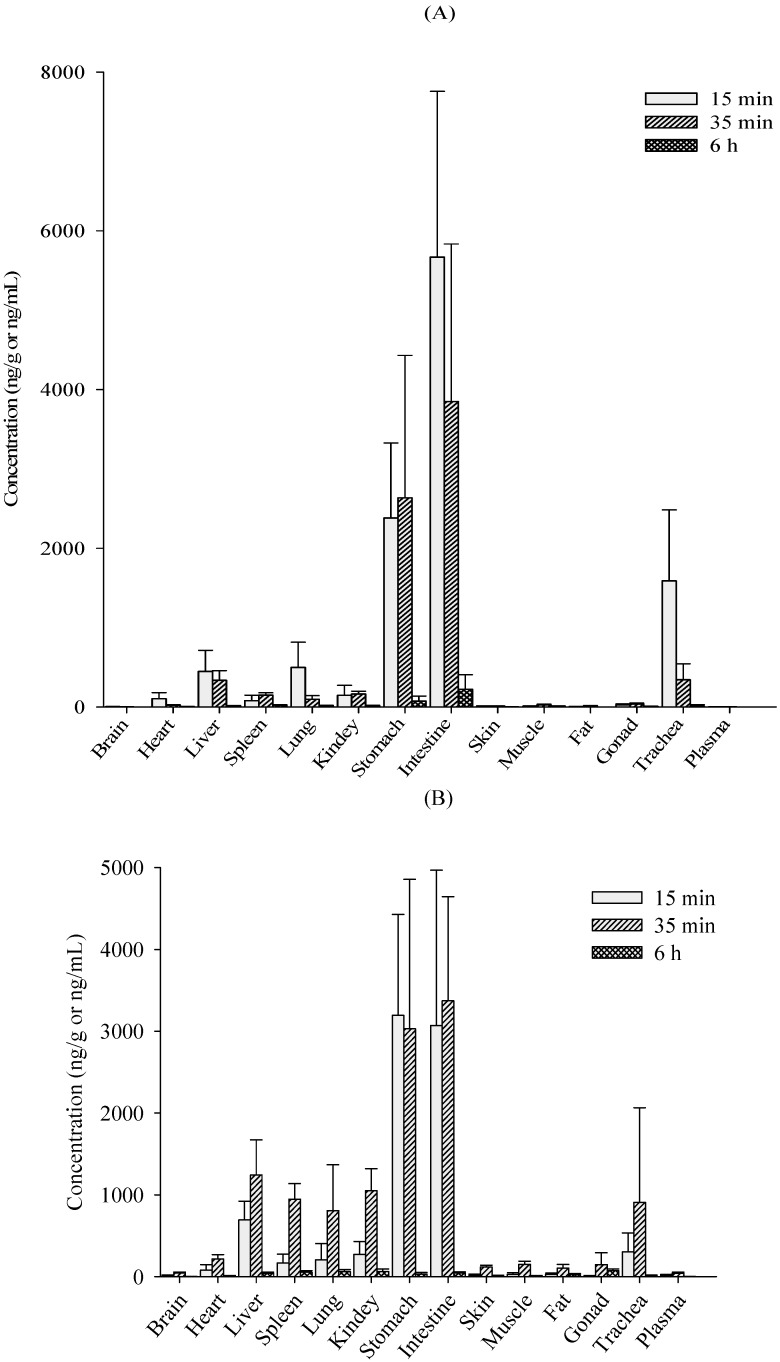
Tissue distribution of verticinone at 15 min, 35 min and 6 h following *i.g.* administration of 2 mg/kg to rats (*n* = 5). (**A**) female; (**B**) male.

The tissue concentrations of verticinone determined at three time points after oral administration (2 mg/kg) are shown in Figure 5. After oral administration, verticinone was rapidly distributed in all organs in rats. A high concentration of verticinone was found in stomach and intestines, whereas very little was found in the brain, which suggested that verticinone had difficulty in passing the blood-brain-barrier (BBB). Six hours after verticinone administration, the concentration of the compound became markedly reduced in all studied tissues, except in gonads of male rats. The AUC values for the individual tissues following *i.g.* administration decreased as follows, intestine > stomach > liver > kidney > spleen > lung > heart > gonad > muscle > skin > fat > brain, in male rats; intestine > stomach > liver > kidney > spleen > lung > gonad > heart > muscle > skin > fat > brain, in female rats.

### 2.3. Plasma Protein Binding Study

The results of the plasma protein binding ability of verticinone* in vitro* of both rats and humans are shown in Table 3. A study of the absorption of ultrafiltration membrane on verticinone showed that it had no absorptive effect on the compound. No saturation of the plasma protein binding was found in a concentration range of 5~100 ng/mL of verticinone in both rats and humans. The plasma protein binding of verticinone was 89.8%~91.3% in rats and 89.5%~94.6% in humans, indicating no significant species difference.

### 2.4. Excretion Study

**Figure 6:**
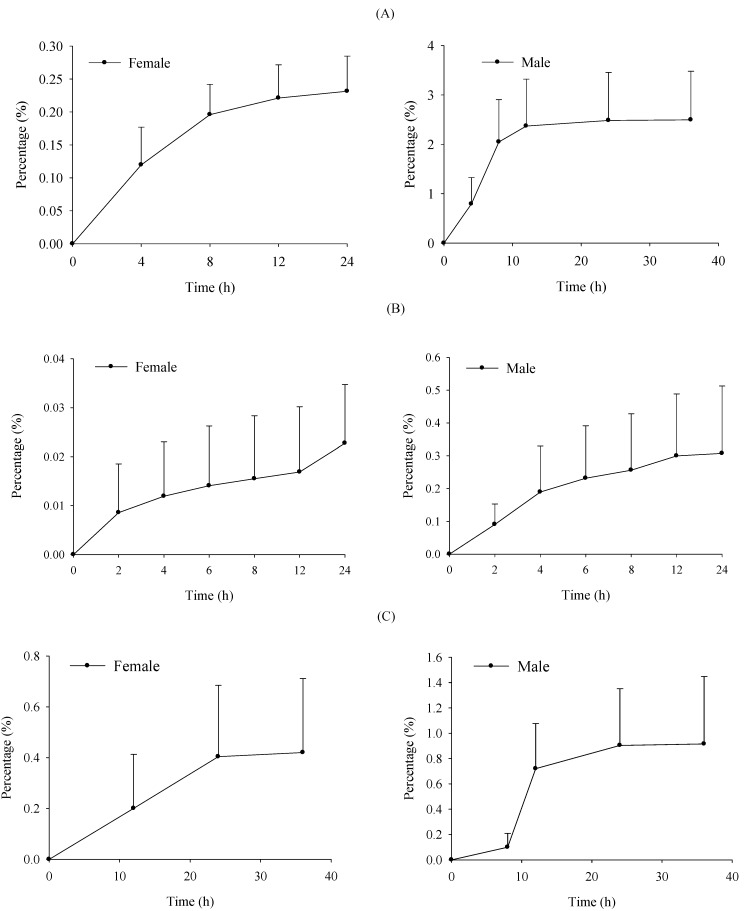
Mean plot of cumulative excretion of verticione following *i.g.* administration of 2 mg/kg to SD rats. (**A**) urine; (**B**) bile; (**C**) feces.

After *i.g.* administration of verticinone, mean plots of cumulative excretion (% of administered dose) in urine, bile and feces are shown in Figure 6. In male rats, cumulative excreted fractions of verticinone in urine (36 h), bile (24 h) and feces (36 h) were 2.50% ± 0.98%, 0.31% ± 0.21% and 0.92% ± 0.53%, respectively, but in female rats, the cumulative excreted fractions of unchanged verticinone in urine (24 h), bile (24 h) and feces (36 h) were only 0.23% ± 0.05%, 0.02% ± 0.01% and 0.42% ± 0.29%, respectively. The recovery from male and female rats feces were both less than 1%, which implied that verticinone may be completely absorbed in rats. The results demonstrated that the lower plasma concentration and urine recovery in female rats may not be caused by absorption. In male rats, the unchanged verticinone excreted from urine and feces was about 3.4%, but in female rats, only 0.6% of unchanged verticinone was eliminated by these routes, which indicated that verticinone was metabolized more extensively in female rats than in male rats.

**Table 3:** The protein binding rates (%) of verticinone in the plasma of rats and humans* in vitro* (Mean ± S.D., *n* = 4).

		5 ng/mL	30 ng/mL	100 ng/mL
Protein binding rates (%)	rats	91.3 ± 2.7	91.0 ± 1.3	89.8 ± 1.1
	humans	92.6 ± 1.0	89.5 ± 0.8	94.6 ± 0.5

### 2.5. Discussion

A significant gender difference in the pharmacokinetics of verticinone in rats was observed when verticinone was administered, both* i.g.* and* i.v.* The clearance of verticinone in rats was higher in females than in males. As a result, the male rats experienced a higher exposure to verticinone than the females when the same dose was administered. No significant difference of t_1/2_ (terminal half-life, which reflects the excretion and metabolism after the finish of absorption) was observed after *i.g.* administration, which indicated that the gender difference may be related to different intestinal absorption, distribution, or first pass effects. The great difference of absolute oral bioavailability between male and female rats further indicated that first pass effects play a significant role in the gender difference in the pharmacokinetics of verticinone. The sex-dependent pharmacokinetics of verticinone was also observed in rats after *Fritillaria thunbergii* Miq. extract was administered [13]. However, the C_max_ and AUC values then were only about 2~3 fold higher in male rats than in females, which indicated that the other constituents in the extract of *Fritillaria thunbergii* Miq. indeed influence the pharmacokinetic behavior of verticinone and the tremendous gender difference was decreased.

Sex differences in pharmacokinetics of xenobiotics that undergo metabolism are not unusual for rats. Gender-specific expression of hepatic CYP isozymes in rats accounts for most cases of gender-related pharmacokinetics of drugs or xenobiotics, in which the drug exposure was lower in male than that in female rats [15,16,17]. However, in the gender-related differences in the pharmacokinetics of verticinone in rats we observed the opposite for the normally reported results. To address this problem, a preliminary metabolism study by LC-IT/TOF-MS method was performed in our lab. The identified metabolites of verticinone in urine, feces and bile indicated that the metabolic pathway for verticinone involved oxidation, reduction, isomerization, sulfate conjugation of the parent drug and its Phase I metabolites. The types of metabolites (mainly Phase I) detected were more in male rats compared to the females. The experiments in rat liver microsomes showed a faster (about 1.7 times) metabolic rate in females (3.76 × 10^−3^ nmol/min/mg protein) than that in males (2.18 × 10^−3^ nmol/min/mg protein). CYP 450 enzymes are the main ones involved in xenobiotics metabolism, some of which show sex different expression. For example, the isoforms CYP 2A1, CYP2C7 and CYP2C12 are female-predominant [18]. However, the exhibited gender difference in phase I metabolism experiment could not explain the tremendous gender differences* in vivo*. Sulfoconjugation has been reported to be gender differentiated in rats. In view of the published reports, there are two types of gender-specific distribution of SULT enzymes in adult rats: the male-predominant phenol-sulfotransferase (SULT1) and the female-predominant hydroxysteroid-sulfotransferase (SULT2) [19]. Liver cytosols from female rats contained 6–8 times as much cortisol sulfotransferase activity as those from males [20,21]. The SULT2 family catalyzes the sulfation of variety of endogenous molecules such as androsterones, glucocorticoids, estrogens, bile acids, and xenobiotics, including steroidal compounds, like budesonide, tibolone, and* etc.* in humans [22,23,24]. PF-02341066, which is a selective c-Met/Alk tyrosine kinase inhibitor currently in clinical development as an anticancer agent, also exhibited gender-related differences in pharmacokinetics with at least 2-fold higher PF-02341066 plasma concentrations in male than in female rats when administered the same dose. The metabolic study of PF-02341066 indicated that the more extensive formation of the parent sulfoconjugate in female rats most likely explains why the female rats had lower drug exposure compared to male rats [25]. Since the sulfate conjugation metabolites of verticinone were found in both male and female rats, considering its similar structure to steroidal compounds, we speculate that sulfoconjugation metabolism might be one of the reasons for the gender-related pharmacokinetics. Considering species differences of drug metabolism between rats and humans, for example, no gender difference in express of SULT2 enzymes in humans has been reported, so it’s unclear whether this tremendous gender difference in pharmacokinetics of verticinone will manifest in humans. To address this problem, a comprehensive metabolism study needs to be done to illustrate the main metabolic route of verticinone in rats.

## 3. Experimental

### 3.1. Chemicals and Reagents

Verticinone (purity > 99%) and hupehenine (purity > 99%) were obtained from the National Institute for the Control of Pharmaceutical and Biological Products (Beijing, China). Methanol, HPLC grade, was purchased from Sigma-Aldrich (St. Louis, MO, USA). Ammonium hydroxide and ethyl acetate were analytical grade. Water was purified with the Milli-Q system (Millipore, Bedford, MA, USA).

### 3.2. Animals

Sprague–Dawley rats (200 ± 20 g) were obtained from the Academy of Military Medical Sciences (Beijing, China). The rats were acclimated to standard housing and environmental conditions (22–24 °C, 60% relative humidity and 12 h light-dark cycle) for one week and fasted for 12 h with free access to water prior to the experiments. All study protocols were approved by the Animal Ethics Committee of China Pharmaceutical University.

### 3.3. Single- and Multiple-Dose Plasma Pharmacokinetics

Pharmacokinetic studies of verticinone after intravenous (*i.v*.) and intragastrical (*i.g.)* administration to rats were designed to determine pharmacokinetic parameters and absolute bioavailability. For* i.g* administration, verticinone was suspended in 0.5% carboxymethyl cellulose sodium (CMC-Na) to final concentrations of 0.2, 0.4 and 0.8 mg/mL. Verticinone was dissolved in 20% ethanol/80% water to final concentration of 0.08 mg/mL for *i.v.* administration. For the *i.v.* group, five male and five female rats received a single dose of 0.4 mg/kg. Blood samples (0.25 mL) were obtained at 0, 4, 15, 30, 45, 60, 120, 240, 360, 480, 600 and 720 min after injection. For the single- *i.g.* group, fifteen male and fifteen female rats were divided into three groups, and each group received a single oral dose of 1, 2 and 4 mg/kg of verticinone, respectively. In addition, another five male and five female rats received multiple oral doses of 4 mg/kg/day, given as 2 mg/kg doses twice a day (one dose in the morning and the second dose 12 h later) for 7 days, then a single oral dose of 2 mg/kg on the eighth day. Blood samples (0.25 mL) were obtained at 0, 5, 15, 30, 45, 60, 90, 120, 240, 360, 480 and 720 min for male rats and at 0, 5, 10, 20, 30, 45, 60, 120, 240, 360, 480 and 720 min for female rats after oral administration (the eighth day for multiple-dose group). Blood samples were then centrifuged at 4000 rpm for 10 min, and the plasma was collected. All plasma samples were frozen and stored at −20 °C until analysis. Verticinone was isolated from plasma by liquid-liquid extraction according to our previously reported procedure [11].

### 3.4. Tissue Distribution Study

A single 2 mg/kg oral dose of verticinone was administrated to thirty rats (15 male and 15 female). Rats were sacrificed and tissues (heart, liver, spleen, lung, kidney, brain, stomach, intestine, skin, muscle, fat and gonads) were promptly removed at 0.25, 0.58 and 6 h (*n* = 10, with 5 male and 5 female) after dosing and washed with saline. Each tissue sample (0.2 g) was homogenized in 5 volumes (*v*/*w*) of distilled water. Tissue homogenates were stored at −20 °C until analysis. Verticinone was extracted from tissue homogenate based on the method described for plasma with slight modifications. The evaporated residue was reconstituted in 200 μL methanol, vortexed, followed by centrifugation at 15,000 rpm twice. An aliquot of 10 μL was injected into the LC-MS system for analysis.

### 3.5. Plasma Protein Binding Study

The ability of verticinone to bind to plasma protein was determined by the ultrafiltration method. A Centrifree Micropartition device (Millipore, Beverly, MA, USA) was used to separate unbound and bound verticinone. Verticinone of different concentrations were added into blank rat and human plasma to give final concentrations at 5, 30 and 100 ng/mL. The spiked plasma was incubated for 30 min at 37 °C and an aliquot (100 μL) was analyzed by previously described methods to determine the total concentration of verticinone. The ultrafiltrate was obtained by centrifuging at 14,000 rpm for 10 min. The concentration of unbound verticinone in filtrate was similarly determined by the previously described methods. The percentage of free fraction (*p*) of verticinone was calculated as follow: *p* = (concentration of verticinone in ultrafiltrate/concentration of verticinone in original spiked plasma sample) × 100%.

### 3.6. Excretion Study

Ten rats were housed separately in metabolic cages and received a single oral dose of 2 mg/kg verticinone. Urine was collected at 0, 4, 8, 12, and 24 h for female rats and at 0, 4, 8, 12, 24, 36 h for male rats. Feces were collected at 0, 12, 24, and 36 h for female rats and at 0, 8, 12, 24, 36 h for male rats. The volume of urine samples was measured before storage at −20 °C. Feces were weighted and homogenized in 5 volumes (*v*/*w*) of 50% methanol/50% water and stored at −20 °C until analysis. In addition, ten rats were anesthetized with ether and then the bile ducts were cannulated with polyethylene tube. The rats also received a single oral dose of 2 mg/kg verticinone. Bile was collected at 0, 2, 4, 6, 8, 12, and 24 h after dosing and stored at −20 °C until analysis. Verticinone was extracted from urine, feces and bile samples based on the method described for tissue samples.

### 3.7. Quantitative Analysis

The quantification of verticinone was performed by a LC-MS method based on the previously reported method with slight modifications [11]. The liquid chromatography system consisted of a model 2010 chromatograph (Shimadzu, Kyoto, Japan) equipped with a Shim-Pack VP-ODS C_18_ column (150 mm × 2.0 mm, 5 μm). A Shimadzu 2010 mass spectrometer (Q-array-Octapole-Quadrupole mass analyzer) equipped with an electrospray ionization interface was used for MS detection. The mobile phase consisted of (A) distilled water and (B) methanol, and the flow rate was 0.2 mL/min. Chromatography separation was achieved using a gradient elution program, as follows: 0.03→6.0 min, B% 15→85; 6.0→7.0 min, B% 85→85; 7.0→8.0 min, B% 85→15; 8.0–12.0 min, B% 15→15. The column and autosampler tray temperature were kept constant at 35 °C and 20 °C, respectively. For the determination of verticinone, the positive mode was selected, along with the conditions as follows.

CDL voltage 25.0 kV, probe voltage 4.5 kV, CDL temperature 250 °C, block temperature 200 °C, gas flow 1.5 L/min. Hupehenine was used as the internal standard (IS). Both verticinone and IS were detected using selected ion monitoring (SIM) at *m/z* 430.25 for verticinone and *m/z* 416.30 for hupehenine (IS), with a dwell time of 200 ms. Blank plasma, tissue homogenate, urine, feces homogenate and bile were used to prepare the corresponding calibration curves to avoid different matrix effects.

### 3.8. Pharmacokinetic and Statistical Analysis

The pharmacokinetic parameters were calculated by the DAS 2.0 software (Chinese Pharmacological Association, Beijing, China) using a standard non-compartmental method. The C_max_ and T_max_ were determined from the observed data. The elimination rate constant (k_e_) was calculated by log-linear regression of verticinone concentrations during the terminal elimination phase and the half-life (t_1/2_) was calculated as 0.693/k_e_. The AUC_0–t_ was calculated by the linear trapezoidal method. The AUC_0–__∞_ was calculated as the sum of AUC_0–t_ and the extrapolated part determined by C_last_/k_e_. The total clearance (CL) was calculated as dose/AUC_0–__∞_. The absolute bioavailability (*F*) was calculated by the following equation: *F* = [(AUC*_i.g_*_._/AUC*_i.v._*) × (Dose*_i.v._*/Dose*_i.g._*)] × 100%. The results were expressed as mean ± SD. Statistical analyses were performed using an unpaired Student’s t-test.

## 4. Conclusions

In summary, verticinone was rapidly absorbed, widely distributed in most tissues, and metabolized extensively before excretion. The pharmacokinetics of verticinone showed significant gender differences in rats. The values of absolute bioavailability in male rats were much higher than in female rats. Verticinone was metabolized more extensively in female rats than in male rats in an excretion study. In the future, a more systematic study including absorption and transport, intestinal and hepatic metabolism* in vivo* and* in vitro* will be done to illustrate the causes of gender difference in the pharmacokinetics of verticinone in rats.
